# Mung Bean (*Vigna radiata*) Treated with Magnesium Nanoparticles and Its Impact on Soilborne *Fusarium solani* and *Fusarium oxysporum* in Clay Soil

**DOI:** 10.3390/plants11111514

**Published:** 2022-06-05

**Authors:** Yasmine Abdallah, Marwa Hussien, Maha O. A. Omar, Ranya M. S. Elashmony, Dalal Hussien M. Alkhalifah, Wael N. Hozzein

**Affiliations:** 1 Department of Plant Pathology, Minia University, Elminya 61519, Egypt; ranya.elashmoni@mu.edu.eg; 2 Department of Soil and Water Analysis, Minia University, Elminya 61519, Egypt; marwa.hussien@mu.edu.eg; 3 Department of Microbiology, Minia University, Elminya 61519, Egypt; mahasaad534@yahoo.com; 4 Department of Biology, College of Science, Princess Nourah bint Abdulrahman University, P.O. Box 84428, Riyadh 11671, Saudi Arabia; dhalkalifah@pnu.edu.sa; 5 Botany and Microbiology Department, Faculty of Science, Beni-Suef University, Beni-Suef 62521, Egypt; hozzein29@yahoo.com

**Keywords:** antifungal activity, MgO, nanoparticles, mung bean, soil health

## Abstract

The nanotechnology revolution is developing daily all over the world. Soil-borne fungi cause a significant yield loss in mung beans. Our study was performed to identify the impact of different concentrations of MgO nanoparticles (MgONPs) and to assess the prevalence of *Fusarium solani* (*F.* *solani*) and *Fusarium oxysporum* (*F.* *oxysporum*) in mung bean plants under in vivo conditions and, subsequently, the remaining impacts on soil health. In vitro studies revealed that MgONPs could inhibit fungal growth. Mung bean plants treated with MgONPs showed a promotion in growth. The obtained MgONPs were applied to the roots of 14-day-old mung bean plants at a concentration of 100 µg/mL. The application of MgONPs at a concentration of 100 µg/mL caused an increase in mung bean seedlings. Compared to the control treated with water, plants exposed to MgONPs at 100 µg/mL showed improvements (*p* < 0.05) in shoot fresh weight (28.62%), shoot dry weight (85.18%), shoot length (45.83%), root fresh weight (38.88%), root dry weight (33.33%), root length (98.46%), and root nodule (70.75%). In the greenhouse, the severity of disease caused by *F. solani* decreased from approximately 44% to 25% and that by *F. oxysporum* from 39% to 11.4%, respectively. The results of this study confirm that the temporal growth of the soil microbial biomass was partially reduced or boosted following the nanoparticle drenching addition and/or plant infections at higher concentrations of 50 and 100 µg/mL while there was no significant decrease at the lowest concentration (25 µg/mL). The current research helps us to better understand how nanoparticles might be used to prevent a variety of fungal diseases in agricultural fields while avoiding the creation of environmental hazards to soil health.

## 1. Introduction

Mung bean (*Vigna radiata* L.), commonly known as green gram, is an essential part of people’s daily diet. It is rich in protein and fiber; thus, it is as an important source of nutrients all over the world. In addition to being a major source of human food, it plays a significant role in maintaining soil fertility by enhancing soil physical properties and fixing nitrogen in soils from the atmosphere [1]. Mung bean is widely cultivated in Asia and is believed to be a native crop from India; it later reached the USA, Australia, and Africa because it is a short-term leguminous crop that can grow successfully under different environmental conditions [2]. Mung bean is suitable as a catch crop in the triple-cropping systems of intensive agriculture in Egypt (Nile Valley and Delta lands) due to its short life cycle (65–75 days). Moreover, it breaks the insect-pest and disease cycle and thereby enhances the sustainability of soil health and overall agroecological farming systems. Twidwel et al. [3] recorded that the delay in the mung bean planting date from May to July produced increased forage, with 2.2 tons/ha. Under Egyptian conditions, Hozayn et al. [4] recorded that some genotypes of mung bean exhibited suitable vegetative growth and yields. The major farming method in Egypt is maize followed by wheat, and this cereal following cereal production strategy has several negative consequences for soil health and water reserves. In addition, the emergence of illnesses and insect pests has steadily raised environmental concerns. As a result, there is a pressing need to replace this cropping pattern with some short- or medium-term leguminous crops to effectively address the difficulties described.

Nanotechnology is one of the most essential technologies in modern science, although few attempts have been made to use it to boost agricultural output. Microorganisms can be developed as bio-nano factories for the production of agriculturally significant particles. Magnesium oxide nanoparticles (MgONPs) show promise as an effective nutrient source for plants, increasing biomass output and nutrient use by stimulating microbial activity [5,6]. Magnesium oxide nanoparticles (MgONPs) have been recognized as safe materials by the United States Food and Drug Administration (21CFR184.1431). Metal nanoparticles, in particular, have been extensively explored; as a consequence, they have been examined and shown to have strong antifungal characteristics [7]. Metal nanoparticles have been produced and utilized to suppress phytopathogenic fungi [8]. Antimicrobial characteristics have been demonstrated for a variety of nanoparticles, including silver nanoparticles, zinc oxide (ZnO) nanoparticles, and titanium oxide (TiO_2_) nanoparticles [9]. However, because of the hazards connected with heavy metal elements and their accumulation in the body, these nanoparticles raise major concerns about their toxicity. MgONPs are an appealing alternative to heavy metal-based nanoparticles such as silver and ZnO because they can be efficiently degraded and metabolized in the body. The released degradation products of Mg^+2^ and OH ions can be effectively eliminated from the body as long as the renal function is normal, eliminating concerns about excessive metal accumulation in the body [10]. Furthermore, the precise mechanism of MgONPs as bactericidal agents and their potential to anticipate plant disease management is yet unknown. Given the potential for nanoparticles to be used in agriculture, it is crucial to note that the growing worry about MgONPs’ toxicity in agroecosystems cannot be overlooked. The antibacterial activity of these nanoparticles and their capacity to impact as few plant cells as possible are intimately connected in all circumstances [11,12].

Current nanomaterials research is focused on determining the effects of nanoparticles on plant development and productivity to improve their application in agricultural production. MgONPs, which have the advantages of being non-toxic, safe, and easy to obtain, are an excellent fungicide against *Fusarium* spp. and show great potential in preventing root and stem infections [13,14]. These findings suggest that MgONPs could be a good alternative to chemical fungicides in crop protection, although more research on their effects on plants, particularly on mung beans, is needed. As a result, understanding the interactions between MgONPs and plants is critical. The underlying mechanism of metal nanoparticles against bacterial infections appears to be the accumulation of reactive oxygen species (ROS) in cells, especially because the created ROS directly impair the cell’s ability to multiply [15]. Microorganism disinfection is thought to be based on direct contact between nanoparticles and biological cells [16]. Considering this, little study has been done on the effects of MgONPs on fungal infections and complex antimycotic systems. Based on past studies, we hypothesized that MgONPs may be antifungal by acting directly on fungal cells [17] based on earlier research. An ideal agricultural microbicide would have no phytotoxicity to plants, which is critical for green and sustainable agriculture. The application of MgONPs as nanoscale fertilizers or light absorption boosters stimulates the growth of numerous crops [18]. Typically, root rot caused by *Fusarium solani* and *Fusarium oxysporum* is widespread around the world and significantly decreases mung bean quality and yield [19,20,21]. Furthermore, despite MgONPs’ strong toxicity against a variety of phytopathogens, concrete evidence for their function in pathogen infection control in vivo is still lacking. As a result, the use of MgONPs as fungicidal agents should be investigated. In this work, the antifungal mechanisms of MgONPs against phytopathogenic fungi were studied in greater depth. These findings provide a new avenue for investigating metal nanoparticles’ substantial potential as a revolutionary disease management method in agricultural applications. The impact of root application of MgONPs on mung bean plants, and its residual influence on soil health in terms of the total count of bacteria and fungus and the soil resistance index, were assessed in this study. The researchers concentrated their attention on how MgONPs affected the growth of mung bean seedlings; to our knowledge, this is the first study to examine the impacts of MgONPs on mung bean plants in depth.

## 2. Results

### 2.1. In Vitro Inhibitory Effect of MgONPs on F. solani and F. oxysporum

**Figure 1:**
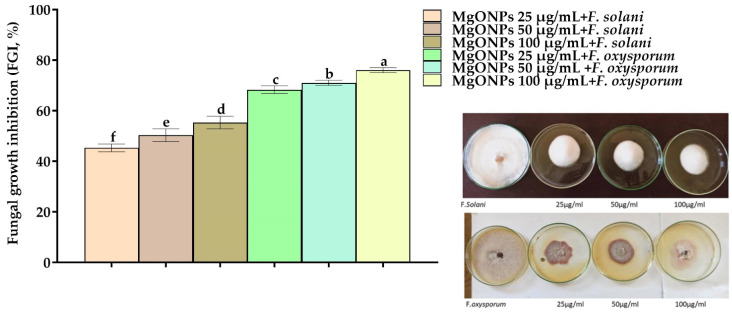
The fungal growth inhibition (FGI%) of MgONPs at different concentrations (25, 50, and 100 µg/mL) on *F. solani* and *F. oxysporum* on NA media. ^a–f^ Columns with different superscripts are significantly different at *p* < 0.05.

The inhibitory effect of MgONPs on the growth of *F*. *solani* and *F*. *oxysporum* in NB media was determined by calculating the percentage of inhibition, as shown in Figure 1. Compared to the control, MgONPs at the final concentrations of 25, 50, and 100 μg/mL showed an inhibition rate of 45.3, 50.3, and 55.3% for *F. solani* and 66, 70.3, and 75%, for *F. oxysporum*, respectively. The suppression of fungal development of *F. solani* in the petri dish was calculated as a percentage compared to the fungus grown in the nutrient media without any treatments. We observed that the lowest concentration was similar to the highest concentration in terms of the impact, and the same was true for the second fungus (*F. oxysporum*).

### 2.2. Impacts of MgONPs on Mung Bean Plants

**Figure 2:**
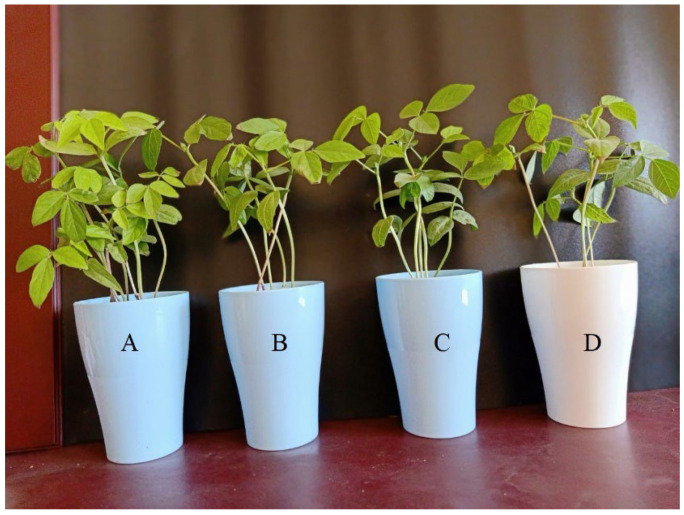
The impact of MgONPs on the growth of mung bean seedlings: A: a concentration of 100 µg/mL; B: a concentration of 50 µg/mL; C: a concentration of 25 µg/mL; and D: only water.

In the absence of the fungal pathogens, the results demonstrated a significantly higher plant growth in mung bean seedlings that were treated by MgONPs (Table 1, and Figure 2). The application of MgONPs at a concentration of 100 µg/mL caused an increase in mung bean seedlings. Comparing to the control treated with water, plants exposed to MgONPs at 100 µg/mL showed significant improvements (*p* < 0.05) in the shoot fresh weight (28.62%), shoot dry weight (85.18%), shoot length (45.83%), root fresh weight (38.88%), root dry weight (33.33%), root length (98.46%), and root nodule (70.75%). Furthermore, in the presence of the fungal pathogens, the plant growth parameters were dramatically improved compared with the previously infected plants. The results suggest that MgONPs can promote the growth of mung bean seedlings in the absence and presence of fungal infection.

### 2.3. The Impact of MgONPs on the Disease Severity of Fusarium solani and Fusarium oxysporum in Mung Bean Seedlings

**Figure 3:**
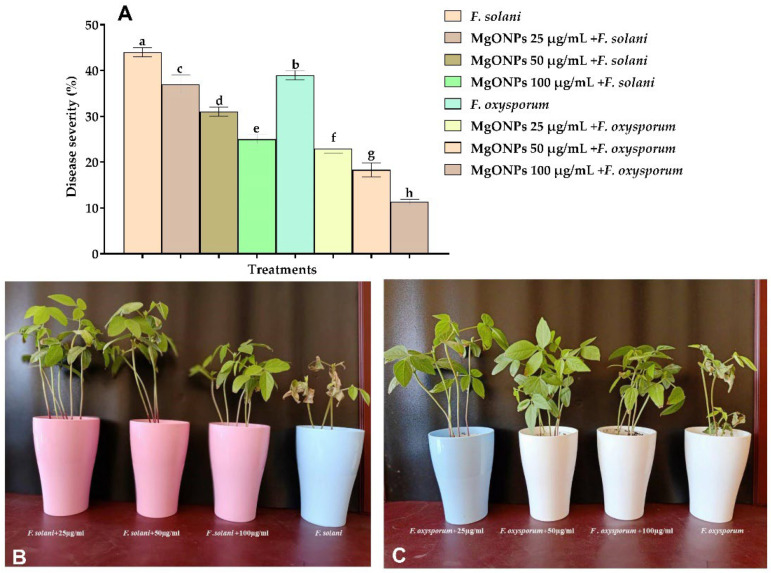
The impact of MgONPs on the disease severity of (**A**) *F. solani* (**B**) and *F. oxysporum* (**C**) in mung bean plants. ^a–h^ Columns with different superscripts are significantly different at *p* < 0.05.

The present study showed MgONPs with strong antifungal activity against the mung bean pathogen of *F. solani* and *F. oxysporum*. The results from Figure 3A–C show the disease severity as a percentage of the mung bean plants reduced to 25% with plants infected with *F. solani* and to 11.33% with plants infected with *F. oxysporum* at a concentration of 100 µg/mL.

### 2.4. Soil Microbial Biomass and Resistance Index after Mung Bean Harvest

The different application rates of MgONPs with and without *Fusarium* plant infection’s impact on the soil microbial biomass counting of bacteria and fungi are shown in Table 2. The trend in the counts of bacteria and fungi among treatments were in the order of control > infection with *Fusarium* alone > foliar application of MgONPs with *Fusarium* infection > MgONPs foliar application alone. The means of the bacteria or fungi counts were significantly higher in the control treatments compared to the *Fusarium* infection alone and MgONPs foliar application alone or the integrated application of both fungi at different concentrations (25, 50, and 100 µg/mL). After soil incubation, significant differences were observed between soil infected with *Fusarium* alone, soil foliar application of MgONPs with *Fusarium* infection, and MgONPs foliar application alone in the counts of bacteria or fungi, reflecting that soil microbiological biomass (SMB) activities were provisionally boosted or rendered by each treatment.

The values of the resistance index (SRI) for soil bacteria and fungi were positive throughout the experiment but differed according to the MgONPs dose and *Fusarium* infection type applied (Table 2). Across all treatments, the soil resistance index (SRI) ranged between 0.448 and 1.00 for bacteria and 0.214 and 1.00 for fungi and was greatest in the control treatment followed by the integrated treatments.

The means of the SRI were significantly higher in treatments with the control compared to all treatments of the MgO-NPs doses and the application of both *Fusarium* infection types. Lower values of SRI indicate an inhibited influence of the treatment type on the microbial biomass activity and lower microbial activity and assimilation balance. Higher SRI values of bacteria and fungi were prominent in the control and MgONPs doses alone, indicating higher microbial activity than the other treatments of the MgONPs doses combined with *Fusarium* infections. The SRI for bacteria and fungi in the MgONPs doses combined with *Fusarium* infection treatments, regardless of *F. oxysporum* or *F. solani*, decreased to a minimal extent and caused stronger disturbances for soil microorganisms than the control and MgONPs doses alone. The temporal effects of the different treatments were more prominent in the counts of fungi than the counts of bacteria for all treatments as indicated by the soil microbial biomass resistance index (SRI).

## 3. Discussion

Nanotechnology products are promising and environmentally friendly strategies for the control of fungal disease. In particular, significant attention has been paid to the development of the plant-based synthesis of metal nanoparticles [22]. Green synthesis has attracted attention for the synthesis of various metal and metal oxide nanoparticles since chemical synthesis techniques result in the presence of harmful compounds adsorbed on the surface of nanoparticles [23]. Metal nanoparticles such as silver are considered the most reported antifungal agents followed by cupper nanoparticles; however, there are still few studies on magnesium nanoparticles that demonstrate the antifungal mechanism. Metallic nanoparticles were used to suppress phytopathogenic increases in our study of the green synthesis of Fe nanoparticles in green and black tea, with concentrations of 102, 550, 100 ppm against *Aspergillus flavus* and *Aspergillus parasiticus* observed in vitro. The results showed a 51.6% inhibition with black tea extract while with green tea extract was 43.5% at 100 ppm [24]. Moreover, another study of Ahmed et al. (2016) [8] on two species of *Fusarium oxysporum* using nickel nanoparticles at a concentration of 50 and 100 ppm in vitro showed that a concentration of 100 ppm significantly reduced mycelial development. Moreover, Ni nanoparticles reduced disease severity in lettuce and tomato plants by 58.4% and 57.0%, respectively, when used in vivo at a concentration of 50 ppm. This study is consistent with the study of Raliya et al. [25], which recorded that up to 250 mg/Kg TiO_2_ promoted the plant height, root length, and biomass. There are a few studies that suggest the positive effect of metal and metal oxide nanoparticles in agriculture, but the mechanisms are mostly unknown, and the data are inconsistent. As a result, much research is required prior to deploying nanoparticles in the field. Most of the research shows that the morphological diversity in plants is a result of metal nanoparticles and metal oxide. A drenching application of MgONPs on tomato plants resulted in significant bacterial wilt in tomato plants [18]. Metal oxide nanoparticles are thought to be a viable alternative for the control of phytopathogenic fungus in agriculture. Several metal nanoparticles (e.g., Ag, Zn, Cu, Au, Se, Ni, Mg, Fe, and Mn) have been produced and tested as antifungal agents. In accordance with our study, the use of Al_2_O_3_ NPs on tomato plants in vitro and in vivo suppressed the fungus *Fusarium oxysporum* [26]. According to the study of Pham et al. [27], Cu nanoparticles’ antifungal activity was determined using tests against *Fusarium* sp. CuNps at a 450 ppm concentration prevented 93.98% of the fungal growth. The study of Akpinar et al. [28] on phytopathogenic *Fusarium oxysporum* f. sp. *radicis-lycopersici* used a treatment of silver nanoparticles (AgNPs) of various nanosizes (3, 5, 8, and 10 nm) and concentrations (12.5–100 ppm). The AgNPs treatment with 3 nm diameters at 25, 37.5, and 50 ppm concentrations inhibited mycelium development by 50%, 75%, and 90%, respectively. The possibility of the antifungal activity of metal nanoparticles could be illustrated as the released ions bind to groups of proteins, which disrupts the function of membrane proteins and results in the permeability of the cell. Moreover, the ions of nanoparticles have a toxic impact on DNA, which causes cell death [16]. The results of this study suggest that the temporal growth of soil microbial biomass may either be partially inhibited or completely facilitated following foliar addition of nanoparticles and *Fusarium* infections, depending on the application rate [29,30,31]. In agreement with the previous study on chickpea (*Cicer arietinum* L), molybdenum nanoparticles enhanced root nodules and promoted the positive activity of microbiological processes in the soil [32].

## 4. Materials and Methods

### 4.1. Preparation of Nanoparticles

**Figure 4:**
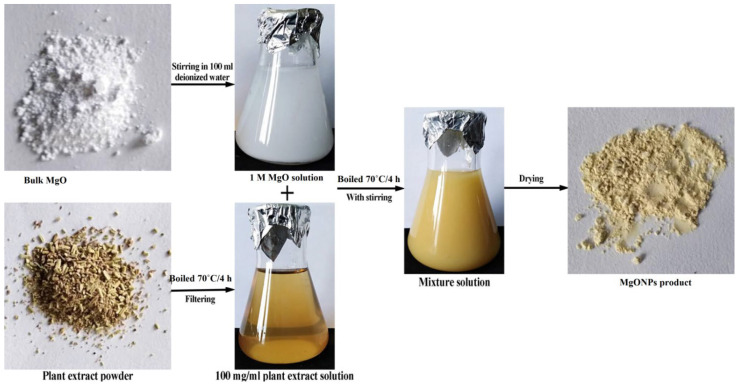
Flowchart diagram of the green synthesis of MgO-NPs using rosemary flower extract (source: [33]).

The green synthesis of MgO nanoparticles using rosemary extract is shown in Figure 4 as previously prepared and characterized through the biological method described by Abdallah et al. [33]. In brief, rosemary flowers were obtained from a local market, and then dried and ground in a domestic blender. The ground flowers were stored under a vacuum and maintained in a domestic freezer at 10 °C. In total, 1 g of ground rosemary was boiled at 70 °C for 4 h with 100 mL of distilled water. The extract was filtered through Whatman filter paper No. 1; then, the yellow-brown extract was collected for further experimental procedures. In total, 100 mL of MgO aqueous solution (1.0 mM) was mixed with 100 ml of rosemary extract and stirred continuously at 600 rpm at 70 °C for 4 h using a magnetic stirrer (Magnetic Stirrer, Jiangsu, China). Then, the mixture was separated by centrifugation at 5000 rpm for 15 min, and the precipitate was washed with distilled water and dried in an ALPHA 1-2/LD-Plus vacuum.

### 4.2. Fungi Associated with Wilted Mung Bean Plants

For this study on mung bean plants, *F*. *solani* and *F*. *oxysporum* were provided by the department of plant pathology, college of agriculture, Minia university.

### 4.3. Isolation of Root Nodule Bacterium

The root nodule bacterium was isolated from root nodules according to Marques-Pinto et al. [34] using yeast extract mannitol Agar [35]. The root nodule bacterium was purified using yeast extract mannitol Agar Supplemented with Congo red as described by Hammad [36]. The isolated root nodule bacterium was kindly identified using the biolog detection technique (Biolog unit EPCRS 018) by Cairo MIRCEN, Fac. Agric., Ain Shams University as *Bradyrhizobium* sp.

### 4.4. Analysis of Soil Properties

The investigated soil had a clay texture and was classified as alluvial soil in accordance with Abd El-Azeim et al. [37]. Previous to the in vitro and greenhouse experimental procedures, the clay soil presented in Table 3 was obtained, then air-dried, sieved to <2.0 mm, and the prepared sample was used to determine the soil properties using the standard methods of Jackson [38], and Page et al. [39].

### 4.5. In Vitro Evaluation of the Antifungal Activity of MgONPs against Fusarium solani and Fusarium oxysporum

#### 4.5.1. Linear Growth

The mycelial growth inhibition (FGI) of *F. solani* and *F. oxysporum* was evaluated using agar diffusion on Nutrient Agar media (NA) media and was sterilized at 121 °C for 15 min. Then, the stock nanoparticle solution was transferred to the NA media to obtain different initial nano MgO concentrations (to make up 25, 50, and 100 µg/mL). The mycelium growth in the PDA medium was measured by inoculating a disk (5 mm in diameter) of 5-day-old fungus in the middle of a dish. Three plates for each treatment were used and then incubated at 25 °C for 7 days in an incubator under dark conditions. After the incubation, the colony diameters were measured in millimeters. Data were recorded by measuring the diameter of the fungal growth (two diameters) and the means where the mycelia growth of any dish reached the edge of the plate. *F. solani and F. oxysporum* were also grown in NA without nanoparticles and evaluated as a positive control:

FGI (%) = (dc − dt/dc) × 100(1)

The percentage of mycelial growth inhibition (FGI) was calculated according to the following formula [40], where dc (mm) is the mean fungal growth diameter for the controls and dt (mm) is the mean fungal growth diameter for each group treated with MgONPs.

#### 4.5.2. Pathogenicity Test

The pathogenic ability of *F. solani* and *F. oxysporum* to induce wilt in mung bean plants was evaluated under greenhouse conditions. The two fungal species were grown separately on autoclaved sorghum grain sand medium (100 g washed dried sorghum grains, 100 g washed dried coarse sand, and 65 mL tap water per bottle) in 500 mL glass bottles. Inoculation was carried out using uniform agar discs (5 mm diameter) bearing 4-day-old fungal growth of any of the tested isolates. The bottles were incubated at 28 °C for 2 weeks to obtain sufficient growth of the fungal isolates. The fertile soil was taken from the surface layer of the soil of the experimental farm Faculty of Agric. Minia Univ. and was sterilized using formalin solution (5%). Formalin disinfested clay pots (30 cm diameter) were filled with sterilized soil at a rate of 3 kg/pot. The potting soil was then artificially infested with the desired inoculum prepared at a rate of 3% (*w*/*w*), and then watered 2 times 1 week before planting. In the check treatments, equal amounts of uninoculated substrate were added to the pots [41]. Mung bean seedlings grown for 30 days in seed boxes filled with autoclaved peat-moss vermiculite (1:1 *w*/*w*) were uprooted and transplanted to the pots at a rate of 3 seedlings/pot. Three replicate pots were used for each fungal isolate. Pots were irrigated directly after transplanting and subsequently when necessary.

#### 4.5.3. Disease Index of Foliar Browning

The degree of foliar yellowing disease was measured by grading each leaf on the severity of wilt symptoms and yellowing on a 0–4 scale and the average grade for the entire plant was calculated using the formula: (Sum of foliar yellowing value/(4 × Total number of the leaves) × 100 = percent of foliar yellowing [42]. In the present study, the following numerical grades were used: 0 = healthy plants; 1 = 1—less than 25% of the plant leaflets are yellow (slight chlorosis, wilting, or stunting); 2 = 25—less than 50% of the plant leaflets are yellow (moderate chlorosis, wilting, or stunting); 3 = 50—less than 75% of the plant leaflets are yellow (severe chlorosis, wilting, or stunting); 4 = 75—less than 100% of the plant leaflets are yellow (very severe chlorosis, complete wilting, or dead plant).

### 4.6. In Vivo Effect of MgONPs on the Development of Vascular Wilt in Mung Bean Seedlings Inoculated with Fusarium oxysporum and Fusarium solani

Plants were harvested four weeks after the soil application to examine the plant’s morphology state. Shoots were cut at the soil surface, and roots were gently shaken to remove extra soil, clumps of soil caught between roots were removed, and the number of nodules and root length were measured.

### 4.7. Soil Incubation Experiment after Mung Bean Harvest

After the mung bean plant harvest, a sample of 1 kg of soil was taken from each experimental pot to be incubated under controlled conditions for 10 days at 30 °C under a 65% soil water holding capacity to estimate the impacts of MgONPs and *Fusarium* infection on soil health in terms of the soil biological properties.

#### 4.7.1. Analyses of Soil Biological Properties

##### Total Bacteria and Total Fungi and Soil Resistance Index (SRI)

After soil aerobic incubation for 10 days, soil samples collected for the determination of soil biological properties were sieved to pass through a 1.5 mm mesh. After the end of the experiment, the plate count technique in accordance with Alef [43] was used to determine the total counts of bacteria and fungi in soil samples. The colony-forming units (CFUs) of total bacteria were counted on nutrient agar while total fungi (CFU) were counted on potato dextrose agar media. The total counts of bacteria or fungi that withstood each treatment were determined using the soil resistance index (SRI) calculated by the equation established by Orwin [44]:

(2) $$\mathrm{SRI}\left(t_{0}\right)=1\;–\;\frac{2\left(D_{0}\right)}{\left(C_{0}+\left[D_{0}\right]\right)},$$

The index anticipated for the soil resistance index (SRI) was calculated as (D_0_) the difference between the untreated and undisturbed soil control (C_0_) and the soil treated with MgONPs or *Fusarium* (F_0_) at different rates at the end of the experiment time (t_0_) (i.e., time 0 or t_0_ at the end of the experiment). This takes into account differences in the amount of change in the soil’s total counts of bacteria and fungi that an added disturbance could cause, considering that the foliar application of MgONPs and/or *Fusarium* infection are soil disturbance factors. This index of soil resistance is between +1 and −1, with +1 indicating the treatment had no disturbance effect (greatest resistance), and inferior data showing stronger effects (low resistance).

### 4.8. Statistical Analysis

Data were subjected to analysis of variance (ANOVA) using SAS 2003 software (SAS Institute, Cary, NC, USA). A general linear model (GLM) procedure was used to check the significant differences among the main treatments. Individual comparisons between the mean values were performed using Duncan’s method (*p* < 0.05).

## 5. Conclusions

The effect of MgONPs on the mung bean plant was revealed in this study. Plant growth was promoted at various concentrations. This study found that 100 µg/mL MgONPs was the most efficient therapy for improving growth among the various concentrations studied. Furthermore, the current study demonstrated the importance of MgONPs in plant disease management. MgONPs exhibited antifungal activity against *F. solani* and *F. oxysporum* in vitro and in vivo successfully suppressed the infection in mung bean plants. Under both in vitro and in vivo conditions, MgONPs were shown to have promising biocontrol potential. In addition, MgONPs are also less damaging to soil microbiomes and health. In this respect, our findings substantiate the use of MgONPs in the treatment of plant diseases, indicating the promising prospects of nanofertilizers and fungicide. However, more study is needed to confirm the effect of MgONPs on several phytopathogens that cause substantial crop losses in the field.

## Figures and Tables

**Table 1:** The impact of MgONPs on the shoot fresh weight, shoot dry weight, shoot length root fresh weight, root dry weight, root length, and the number of root nodules of mung bean seedlings.

Treatments	Shoot Fresh Weight (g)	Shoot Dry Weight (g)	Shoot Length (cm)	Root Fresh Weight (g)	Root Dry Weight (g)	Root Length (cm)	Root Nodule (n)
Control	5.10 ± 0.08 ^c^	0.81 ± 0.01 ^d^	24.00 ± 0.73 ^e^	1.80 ± 0.08 ^d^	0.60 ± 0.08 ^e^	9.10 ± 0.08 ^ed^	8.00 ± 0.81 ^fg^
MgONPs 25 µg/mL	5.50 ± 0.32 ^c^	1.03 ± 0.12 ^c^	27.66 ± 0.47 ^c^	2.00 ± 0.08 ^c^	0.65 ± 0.01 ^c^	10.86 ± 0.18 ^cd^	11.66 ± 0.47 ^d^
MgONPs 50 µg/mL	5.93 ± 0.73 ^b^	1.12 ± 0.08 ^b^	30.00 ± 0.81 ^b^	2.21 ± 0.01 ^b^	0.71 ± 0.08 ^b^	14.20 ± 0.21 ^b^	13.33 ± 0.47 ^b^
MgONPs 100 µg/mL	6.56 ± 0.49 ^a^	1.50 ± 0.01 ^a^	35.00 ± 0.81 ^a^	2.50 ± 0.08 ^a^	0.80 ± 0.01 ^a^	18.06 ± 0.17 ^a^	13.66 ± 0.47 ^a^
*F. solani*	2.43 ± 0.09 ^g^	0.313 ± 0.09 ^i^	14.5 ± 0.40 ^i^	0.75 ± 0.04 ^g^	0.25 ± 0.08 ^i^	4.80 ± 0.08 ^g^	3.33 ± 0.47 ^i^
MgONPs 25 µg/mL + *F. solani*	3.80 ± 0.08 ^e^	0.586 ± 0.01 ^g^	20.16 ± 0.62 ^g^	1.59 ± 0.04 ^e^	0.50 ± 0.08 ^g^	8.00 ± 0.12 ^e^	7.66 ± 0.47 ^g^
MgONPs 50 µg/mL + *F. solani*	3.91 ± 0.08 ^e^	0.650 ± 0.09 ^f^	22.00 ± 0.81 ^f^	1.70 ± 0.08 ^ed^	0.55 ± 0.04 ^f^	9.03 ± 0.04 ^ed^	8.33 ± 0.47 ^f^
MgONPs 100 µg/mL + *F. solani*	4.20 ± 0.08 ^de^	0.680 ± 0.01 ^ef^	24.00 ± 0.40 ^e^	1.74 ± 0.04 ^cd^	0.59 ± 0.01 ^ef^	9.76 ± 0.08 ^d^	9.33 ± 0.47 ^e^
*F. oxysporum*	3.00 ± 0.16 ^f^	0.41 ± 0.01 ^h^	18.00 ± 0.40 ^h^	1.10 ± 0.08 ^f^	0.30 ± 0.04 ^h^	5.10 ± 0.08 ^f^	4.00 ± 0.81 ^h^
MgONPs 25 µg/mL + *F. oxysporum*	4.36 ± 0.12 ^de^	0.700 ± 0.01 ^e^	22.00 ± 0.62 ^f^	1.77 ± 0.04 ^cd^	0.60 ± 0.01 ^e^	10.00 ± 0.32 ^d^	9.34 ± 0.47 ^e^
MgONPs 50 µg/mL + *F. oxysporum*	4.50 ± 0.08 ^de^	0.730 ± 0.01 ^ed^	25.00 ± 0.81 ^ed^	1.82 ± 0.08 ^cd^	0.63 ± 0.04 ^d^	11.00 ± 0.08 ^c^	11.33 ± 0.47 ^d^
MgONPs 100 µg/mL + *F. oxysporum*	4.70 ± 0.08 ^d^	0.753 ± 0.01 ^ed^	26.00 ± 0.81 ^d^	1.91 ± 0.08 ^cd^	0.64 ± 0.01 ^d^	11.50 ± 0.08 ^c^	12.66 ± 0.47 ^c^

^a–i^ Columns with different superscripts are significantly different at *p* < 0.05.

**Table 2:** Total counts of bacteria and fungi and the soil resistance index (SRI) as impacted by different treatments.

Soil Resistance Index (SRI) and Total Counts of Bacteria and Fungi				
Treatment	Total Counts of Bacteria (×10^6^ cfu g^−1^)	SRI	Total Counts of Fungi (×10^4^ cfu g^−1^)	SRI
Control	62.63 ^a^	1.00	46.30 ^a^	1.00
MgONPs 25 µg/mL	59.87 ^a^	0.926	44.73 ^ab^	0.935
MgONPs 50 µg/mL	44.60 ^cd^	0.553	26.23 ^d^	0.395
MgONPs 100 µg/mL	38.77 ^e^	0.448	16.33 ^e^	0.214
*F. solani*	47.23 ^c^	0.605	38.77 ^bc^	0.720
*F. oxysporum*	55.23 ^b^	0.789	40.03 ^abc^	0.762
MgONPs 25 µg/mL + *F. solani*	40.70 ^de^	0.481	19.83 ^de^	0.273
MgONPs 50 µg/mL + *F. solani*	45.97 ^cd^	0.580	37.67 ^c^	0.686
MgONPs 100 µg/mL + *F. solani*	46.33 ^c^	0.587	33.97 ^c^	0.579
MgONPs 25 µg/mL + *F. oxysporum*	47.23 ^c^	0.622	39.17 ^bc^	0.731
MgONPs 50 µg/mL + *F. oxysporum*	54.73 ^b^	0.779	41.13 ^abc^	0.757
MgONPs 100 µg/mL + *F. oxysporum*	46.23 ^c^	0.603	37.77 ^bc^	0.710
L.S.D _0.05_	7.331	0.304	7.512	0.321

^a–e^ Columns with different superscripts are significantly different at *p* < 0.05.

**Table 3:** Estimated soil physicochemical characteristics.

Soil Property			
Soil Chemical Properties		Soil Physical Properties	
pH (1:2.5 water)	7.7	F.C%	42.45
EC (dS m^−1^ at 25 °C)	1.35	PWP%	13.78
CEC (cmol_c_ kg^−1^)	37.87	WHC%	48.76
O.M (g kg^−1^)	28.61	A.V (F.C-PWP) %	28.67
Total N (g kg^−1^)	1.29	Clay (%)	56.45
Total C/N Ratio	22.18	Sand (%)	17.76
S.O.C g kg^−1^	18.48	Silt (%)	25.79
Total P (g kg^−1^)	0.56	Soil texture	Clay

FC = field capacity, WHC = water holding capacity, PWP = wilting point, AV = available water, SOC = soil organic carbon, PWP = permanent wilting point, RS = resistance index, EC = electric conductivity, CEC = cation exchange capacity and OM = organic matter.

## Data Availability

The data presented in this study are available on request from the corresponding author.
